# 

**Published:** 1886-08

**Authors:** 


WHOLE NUMBER CCCXIII.

AUGUST, 1886.

NUMBER 1. VOLUME XXVII.

### THE BUFFALO Medical AND Surgical Journal

ESTABLISHED IN YEAR 1844.

**EDITORS:**

**THOS. LOTHROP, M. D.** **A. R. DAVIDSON, M. D.**

ASSOCIATE EDITORS:

FLOYD S. CREGO, M. D. CARLTON C. FREDERICK, M. D.

FRED. B. CAMPBELL, M. D. HERBERT MICKLE, M. D.

EDWARD B. ANGELL, M. D., Rochester, N. Y.

Entered at the Post-Office at Buffalo, N. Y., as Second-class Mail Matter.

